# Novel Allosteric Sites on Ras for Lead Generation

**DOI:** 10.1371/journal.pone.0025711

**Published:** 2011-10-25

**Authors:** Barry J. Grant, Suryani Lukman, Harrison J. Hocker, Jaqueline Sayyah, Joan Heller Brown, J. Andrew McCammon, Alemayehu A. Gorfe

**Affiliations:** 1 Center for Computational Medicine and Bioinformatics, University of Michigan, Ann Arbor, Michigan, United States of America; 2 Department of Chemistry and Biochemistry, Center for Theoretical Biological Physics and Howard Hughes Medical Institute, University of California San Diego, La Jolla, California, United States of America; 3 Bioinformatics Institute, Agency for Science, Technology and Research, Singapore; 4 Department of Chemistry, University of Cambridge, Cambridge, United Kingdom; 5 Department of Integrative Biology and Pharmacology, University of Texas Health Science Center at Houston, Houston, Texas, United States of America; 6 Department of Pharmacology, University of California San Diego, La Jolla, California, United States of America; The University of Kansas Medical Center, United States of America

## Abstract

Aberrant Ras activity is a hallmark of diverse cancers and developmental diseases. Unfortunately, conventional efforts to develop effective small molecule Ras inhibitors have met with limited success. We have developed a novel multi-level computational approach to discover potential inhibitors of previously uncharacterized allosteric sites. Our approach couples bioinformatics analysis, advanced molecular simulations, ensemble docking and initial experimental testing of potential inhibitors. Molecular dynamics simulation highlighted conserved allosteric coupling of the nucleotide-binding switch region with distal regions, including loop 7 and helix 5. Bioinformatics methods identified novel transient small molecule binding pockets close to these regions and in the vicinity of the conformationally responsive switch region. Candidate binders for these pockets were selected through ensemble docking of ZINC and NCI compound libraries. Finally, cell-based assays confirmed our hypothesis that the chosen binders can inhibit the downstream signaling activity of Ras. We thus propose that the predicted allosteric sites are viable targets for the development and optimization of new drugs.

## Introduction

Ras proteins are key regulators of signaling pathways controlling normal cell proliferation and malignant transformation. Signal propagation through Ras is mediated by a regulated GTPase cycle that leads to active and inactive conformations, which differ significantly in their affinity for downstream effectors. Somatic point mutations that perturb the fidelity of this cycle can lead to constitutively active oncogenic Ras [1]. Such mutants are found in about a third of all human tumors where they contribute to the deregulation of cell growth, tumor invasiveness and new blood vessel formation [2]. Germline Ras mutations are also frequently expressed in patients suffering from a group of related developmental disorders, referred to collectively as neuro-cardio-facial-cutaneous syndrome [3], [4], [5]. These disorders share a variable degree of mental retardation, cardiac defects, craniofacial dysmorphism, and short stature [6]. Therapies that target Ras proteins and the signaling pathways under their regulations are thus of major importance for human health.

Considerable effort has been directed towards inhibiting Ras processing enzymes and major components of Ras signaling pathways. Inhibitors of farnesyl and palmitoyl transferases [7] have been investigated for their potential to attenuate C-terminal lipid modification of Ras required for correct plasma membrane localization and subsequent signaling. A drawback of such inhibitors is their poor selectivity as they likely affect many lipid-modified proteins. Furthermore, the most promising farnesyltransferase inhibitors failed to achieve their intended goal of disrupting Ras membrane-binding [8]. Indeed the most frequently mutated Ras isoforms in human tumors (K-Ras and N-Ras) were found to undergo alternative prenylation and remain oncogenically active [9]. Attempting to inhibit the function of K-Ras and N-Ras by using a combination of prenylation inhibitors failed because of the very high toxicity associated with the required combination therapy [10]. Indeed, it is likely that the lack of toxicity associated with farnesyltransferase inhibitors in isolation is due to their inability to inhibit the functions of the endogenous Ras isoforms essential for normal cell viability. Another approach has involved inhibitors of the upstream protein kinase regulators and downstream effectors of Ras, for example, receptor tyrosine kinase inhibitors and components of the RAF-MAPK pathway [11], [12]. However, because Ras proteins are activated by a myriad of stimuli and utilize a multitude of downstream effectors, a particular kinase inhibitor will likely impair only a subset of Ras functions leading to potentially limited therapeutic benefits.

The development of small-molecule inhibitors that directly target Ras is highly desirable but has proven to be a major challenge. Notable issues include the limited bioavailability of drugs that target highly polar active sites, such as the nucleotide-binding site of Ras, also known as the switch region [13]. Additional selectivity and toxicity issues arise from the highly conserved nature of this switch region across small G-proteins and the wider P-loop NTPase superfamily. Furthermore, Ras signaling involves a tightly regulated network of multiple positive and negative regulators with a specific spatiotemporal organization on cellular membranes [2]. It is the balance of these positive and negative regulators that ultimately determines the fraction of GTP-bound active and GDP-bound inactive Ras. Currently it is not clear if normal and aberrant Ras have distinct plasma-membrane organizations that can lead to differential accessibility to downstream effectors and/or upstream exchange factors. These complications may explain, at least in part, why Ras-binders that have promising anti-cancer activity in pre-clinical models failed in clinical trials [14].

**Figure 1:**
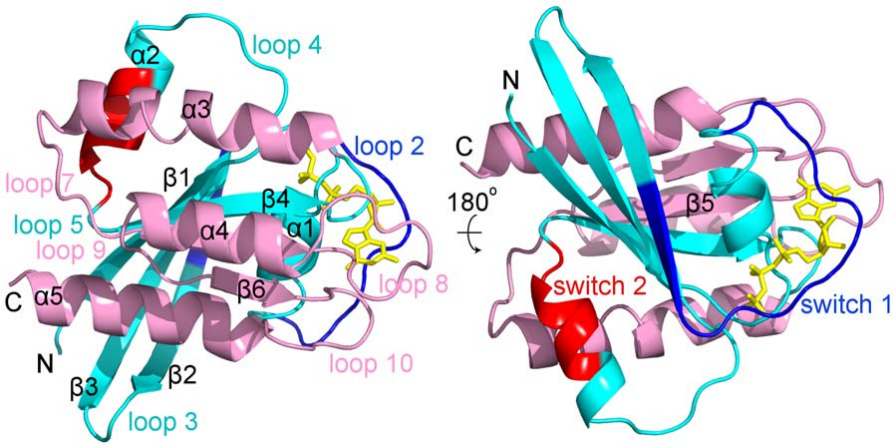
The N-terminal (cyan) and the C-terminal (pink) lobes of K-Ras (PDB code: 2PMX). The switches 1 (blue) and 2 (red), that form the nucleotide-binding site, are situated in the N-terminal lobes. GTP (yellow) is shown as sticks.

The development of compounds with selectivity for K-Ras over H-Ras would be particularly desirable. Silencing of K-Ras by siRNA [15], [16], miRNA [17], [18] or antisense K-Ras [19] has been shown to result in reversal of transformed phenotypes and suppression of tumorigenicity in human cancer cells. Studies of the three major H-, K-, and N-Ras isoforms [20] suggest that differential membrane-organization may underlie the association of these highly similar proteins with different diseases [1]. Similarly, recent evidence from experimental [21], [22] and computational efforts [23], [24] suggests that oncogenic and normal Ras proteins harbor distinct dynamic properties that may lead to differences in membrane binding [25]. Of special note are long-range coupled motions between the conserved N-terminal lobe1 (residues 1–86, which includes the switch region) and the more variable membrane-interacting lobe2 [23] (**Fig. 1**). Such dynamic behaviors are not always captured in crystallographic structures. For example, while the overall structures of wild-type H-Ras and the oncogenic variant G12V-H-Ras are very similar, the latter exhibits enhanced dynamics [26] and is more susceptible to adopt an active-like conformation [23]. Here we ask if such motional differences can be exploited to develop new allosteric inhibitors that selectively target a specific malfunctioning Ras protein.

In subsequent sections we describe the application of an integrated bioinformatics approach that couples fragment, grid and ligand based binding-site mapping methods with virtual screening (VS) of small molecule libraries against an ensemble of distinct Ras conformers. Four non-nucleotide binding pockets were identified in available crystal structures and molecular dynamics simulated conformers. Simulations reveal that three of these distal pockets have the potential to be allosterically linked to the switch region. Furthermore, a number of lead compounds predicted to bind these allosteric sites were tested experimentally and found to exhibit inhibitory activity in Ras-expressing cancer cells.

## Results and Discussion

### A structural ensemble scheme to account for conformational diversity

Conformational transitions are essential for modulating Ras signaling activity. These transitions have the potential to expose transient surface pockets and allosteric sites of significance for drug discovery. Indeed the merit of accounting for receptor flexibility in ligand design [27], [28], [29], [30], [31], especially in the development of allosteric inhibitors [32], [33], is now well recognized. In the current work, we combine analysis of multiple conformationally distinct crystallographic structures with molecular dynamics (MD) trajectory analysis. This ensemble approach differs from earlier virtual screening studies on single crystallographic structures [34], [35] by accounting for the intrinsic conformational mobility of Ras proteins.

Ras crystal structure representatives were selected based on a previously developed protocol for the analysis of inter-conformer relationships in families of protein structures [23], [24], [36], [37]. Principal component analyses (PCA) and RMSD based clustering of available structures indicate the existence of distinct Ras conformational groupings (**Fig. S1**). It has been noted previously that the PCA based groupings correlate with the nature of the bound nucleotide or the presence of a mutation in the vicinity of the nucleotide-binding site [23], [24]. Considering these differences in bound nucleotide and mutation, as well as isoform, seven representative structures, termed the crystallographic ensemble, were used for further analysis (**Table S1**). Additional conformers were derived from MD simulations performed on the K-Ras (the most frequently mutated isoform in a wide range of cancers [38], [39]). Conformers from a total of 120 ns multi-copy simulations performed in the presence of GTP and GDP were subjected to PCA and RMSD clustering analysis similar to that performed on the available crystal structures (see Methods). In this manner, eight representative MD ensemble conformers were appended to the crystallographic ensemble and used for binding site mapping and small molecule docking studies described below.

### Ensemble binding site mapping

A combination of fragment, grid and ligand based methods was employed to identify potential small molecule binding sites on our ensemble of Ras structures. The common practice in computer aided structure based drug design is to aim for inhibitors that compete with an enzyme's cognate substrate [40]. However, in the current work, we deliberately focused on the identification of potential allosteric small molecule binding sites that are distal from the nucleotide-binding site (see Introduction). Taking the union of identified sites from a combination of different methods increases our chances of mapping all such sites at the expense of considering potentially more spurious sites [41].

### Fragment based pocket analysis

**Figure 2:**
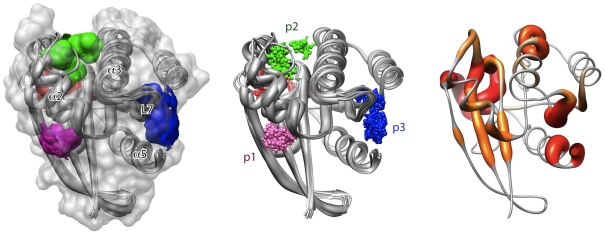
Results of ensemble fragment mapping analysis. Sites identified include the nucleotide-binding site (red) and new potential binding sites p1, p2 and p3 (in pink, green and blue respectively, see also Fig. 3).

**Figure 3:**
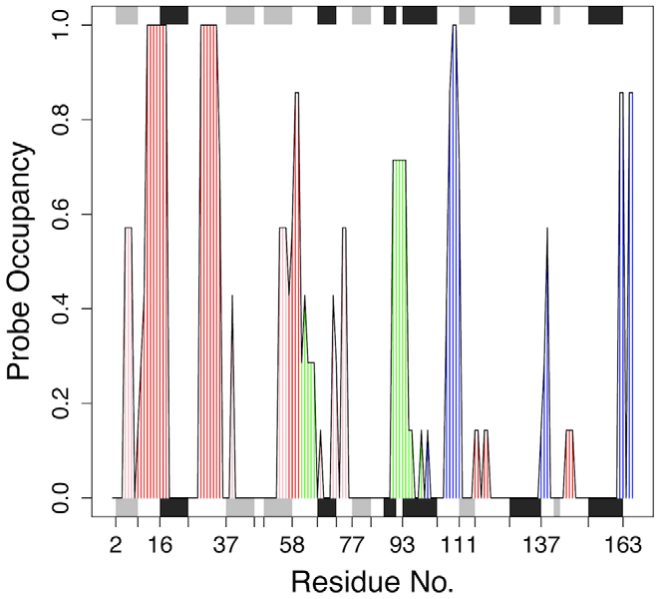
Probe binding “hot-spot” residues across the Ras ensemble conformers highlight the nucleotide-binding site (red) and new potential binding sites p1, p2 and p3 (in pink, green and blue respectively, see also **Fig. 2**).

We first used a fragment based approach (FTMAP) that is based on the ideas behind screening small organic fragments by NMR and X-ray crystallography [42]. FTMAP correlates pocket druggability with their propensity to bind clusters of small organic compounds. **Fig. 2** displays the mapping results across our ensemble of Ras crystal structures. To further characterize the location of each hot spot, an ensemble normalized probe occupancy value was calculated and assigned to each residue of the catalytic domain (**Fig. 3**). As expected, high probe occupancy values were obtained for residues around the nucleotide-binding site (red in **Figs. 2** and **3** and **Movie S1**), consistent with the existence of this binding site in all conformers. Three additional pockets, displaying low and high occupancies, were also identified (termed p1, p2 and p3 and rendered in pink, green and blue respectively in **Figs. 2** and **3** and **Movie S1**).

Sites with low probe occupancy values identify regions on the protein surface where potential interaction sites are exposed only in a subset of conformations (reflecting the intrinsic dynamic nature of these binding pockets). This includes pockets p1 and p2 that reside on either side of the highly mobile helix α2. Residues from α2 and β-strands 1–3 (in lobe1) line p1, whilst p2 lies between helices α2 and α3 at the interface between the two lobes (see **Table 1**). The large relative displacement of α2 between GDP- and GTP- like conformers effectively modulates the accessibility of these two pockets. These nucleotide-associated displacements effectively link the conformations of p1 and p2 to that of the nucleotide-binding site. Of particular note is Tyr71 that lies at the center of pocket p1 blocking ligand binding in the crystal structures of GDP-H-Ras and H-Ras-G12V. During simulations this residue is observed to reorient so as to no longer block p1 pocket accessibility (**Fig. S2**).

**Table 1:** Results of pocket identification on the Ras structural ensemble through fragment (FTMAP), grid (AutoLigand) and ligand (BlindDock) based site identification schemes.

Pocket	FTMAP	AutoLigand	BlindDock	Location	Mean volume
P1	5–7,39,54–56, 67, 70–75	5,7,39,54–56,70–72,74–75	N.A.	β1–3 and α2	112 (78.6)
P2	61–65, 90–99	N.A.	N.A.	L2, α2 and α3	143.6 (120)
P3	97,101,107–111, 136–140, 161–166	97,101,107–111,137–140, 161–163,165–166	97,101,107–112, 137–140,159,162, 163,165, 166	L7, L9 and α5	173 (18.7)
P3b	N.A.	75–78,104,106–110, 162–166	N.A.	L5, L7 and α5	36 (2.1)
P4	N.A.	N.A.	24–40, 17, 21, 57	L2, α1 and β2	139.9 (57.6)

Location specifies surrounding 2° structure elements. Mean pocket volume and standard deviation across the ensemble is calculated with the Povme program [63] (see methods).

The most distal non active site pocket, p3, resides in lobe2 approximately 25 Å from the nucleotide-binding site. Residues from loop 7 and the C-terminal end of helix 5 line this pocket which has a mostly hydrophobic interior but is surrounded by polar residues K104, S106, D108 and H166. Pocket p3 can vary in size in the ensemble, due predominately to small displacements of loop 7 residues. Indeed loop7 and helix 5 are observed to adopt distinct conformations in the GTP-, GDP-, and intermediate ensemble structures. However, pocket p3 is observed in all ensemble conformers in contrast to pockets p1 and p2 that are inaccessible in some members of the ensemble. Considering the dynamic nature of loop 7, α5 and α2 that line pockets p1–3, we hypothesized that VS with our ensemble of conformers may allow us to find new ligand poses that would not be observed with any single crystallographic structure. Finally, we note that the loop 7 comprising pocket p3 has been shown to undergo correlated motions with the active site [23], [24], [43].

### Grid energy based pocket analysis

**Figure 4:**
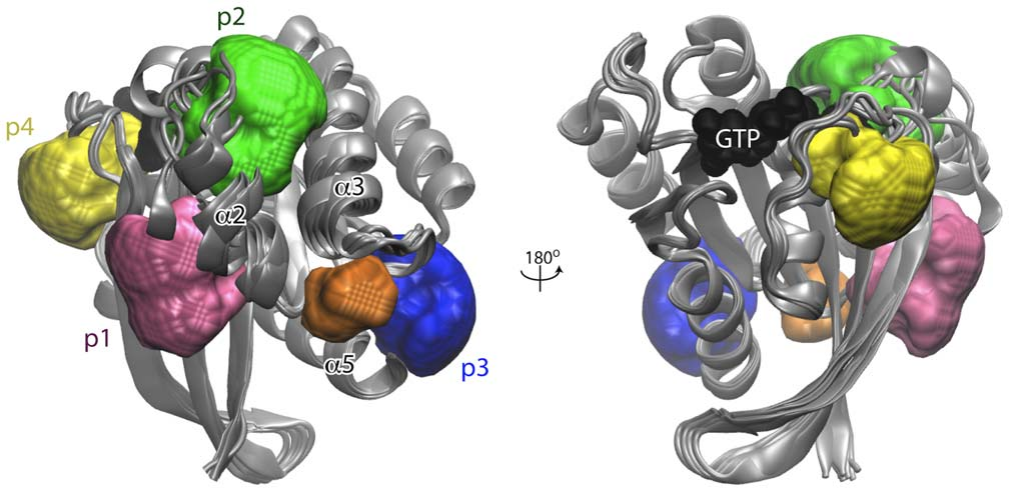
Potential small-molecule binding sites identified on the Ras structural ensemble. The residues lining each pocket are listed in Table 1. The color scheme for each pocket is as in the Figure 2, with p3b (orange) and p4 (yellow) additions.

In addition to fragment based mapping we employed grid energy based pocket identification with the AutoLigand package [44]. AutoLigand searches the exterior surface space of a protein for contiguous volume envelopes with potentially favorable interaction energies for small molecules. Applying this method across the Ras structural ensemble highlighted two of the three previous FTMAP identified binding sites, p1 and p3 (**Table 1**). An additional pocket, here termed p3b was identified immediately adjacent to pocket p3. Both pockets p3 and its extension p3b share residues from loop 7 and the C-terminal end of helix α5. However p3b extends toward loop 5 and p3 toward loop 9 (**Fig. 4**). Pocket p3b is rich in charged residues including E162 that provides a negatively charged site at the bottom of the p3b pocket. Pocket p3b is significantly smaller and shallower than pockets p1–3 as highlighted by FTMAP (**Table 1** and **Fig. 4**), which likely explains why it did not feature in fragment based (described above) or ligand based (described below) site identification.

### Ligand based pocket analysis

As a final step in our characterization of potential binding sites we performed a blind docking of known small-molecule Ras binding molecules. The basic premise of this approach is that a ligand frequently targets a given site if that site offers an energetic or steric advantage over any other site [45], [46]. A library of putative ligands that were shown to bind to Ras at unknown sites from high throughput bioassay data were “blind-docked” onto the entire surface of a single K-Ras crystal structure (see methods). By counting the number of compounds that target a given site, weighted by the frequency with which a given compound targets the site, we found two prominent binding sites (**Table 1** and **Fig. 4**): one that coincides with pocket p3 (targeted by ∼80% of compounds) and a second novel pocket, p4, behind the flexible effector binding loop (targeted by ∼3% of the compounds). To ensure that this dramatic preference for pocket p3 is not biased by the choice of a specific small molecule set, we blind-docked 20 top-ranking compounds derived from a site-directed VS of the NCI and ZINC libraries against the entire crystallographic- and MD-ensembles (to be discussed). We found that these compounds tend to preferentially bind pocket p3, and to a lesser extent to p4. For instance, of the top-ranking poses, 12 out of the 14 ZINC compounds bind to p3 and the other 2 at p4. Similarly, 2 out of the 6 NCI compounds preferentially target p3 while the remaining 4 target pocket p4. Overall, it is noteworthy that three distinct procedures applied to a different set of ligand libraries and fragment probes highlighted the same potential pockets.

### Ensemble virtual screening against the novel binding sites

Compounds from the NCIDS II and Zinc drugs-now subset were docked against the identified pockets in each of our ensemble structures using the Schrödinger Glide package [47], [48]. We next re-scored compounds ranked as top 20 using eight different scoring functions (see Methods). A total of 195 and 256 top scoring unique compounds were obtained from the screening against the crystallographic and MD ensemble, respectively. A total of 56 of the top-scoring compounds (ranked within the top 20) were able to target the same site in both the crystallographic- and MD-ensembles. The MD-ensemble yielded 26 additional compounds not detected in the screening against the crystallographic-ensemble. Furthermore, some compounds that scored poorly in the crystallographic-ensemble scored well in the MD-ensemble. Such an improvement of the rankings and the appearance of 26 additional compounds as top scorers in the MD-ensemble is largely due to conformers that lie in-between the major GTP- and GDP-clusters (**Fig. S3**). This result highlights the benefit of utilizing the MD-based relaxed complex scheme in addition to the traditional approach of relying on one or a few average X-ray structures [29], [49]. It is important to also note that the crystallographic ensemble is dominated by H-Ras structures (both wild-type and mutant), whereas the MD-ensemble contains only K-Ras structures. It is possible that some of the 26 ligands that appear only in the MD-ensemble may be K-Ras-selective. For subsequent analysis, we focused on the 56 compounds that scored high in both the crystallographic- and MD-ensembles as well as the 26 compounds that are unique to the MD-ensemble. After Lipinski-filtering and visual inspection 19 ligands were selected as promising leads and submitted for experimental testing (**Table 2**).

**Table 2:** Ensemble docking hits selected for experimental testing.

Compund ID	Ligand dataset	Potential Pocket	nOHNH	nON	LogP	MW
16195481	PubChem Bioassay	p3	4	8	1.626	420.52
5446021	PubChem Bioassay	p3	3	7	2.567	397.46
5446018	PubChem Bioassay	p3	3	7	1.882	397.46
5295758	PubChem Bioassay	p3	1	6	3.57	415.56
4121863	PubChem Bioassay	p3	3	5	4.83	392.53
3227807	PubChem Bioassay	p3	1	8	3.737	447.52
12971189	Zinc drugs-now subset	p3	3	6	0.515	228.26
16958504	Zinc drugs-now subset	p3	4	9	−3.901	305.32
17047255	Zinc drugs-now subset	p3	7	6	−0.524	279.33
19166944	Zinc drugs-now subset	p3	1	5	−1.04	312.37
24983237	Zinc drugs-now subset	p3	4	9	−4.469	335.37
6682086	Zinc drugs-now subset	p3	3	5	−0.247	300.38
6691859	Zinc drugs-now subset	p3	7	6	−3.477	185.26
13616	NCIDS II	p3	2	4	4.317	327.46
23895	NCIDS II	p3	2	4	2.857	221.25
36818	NCIDS II	p3	2	8	1.602	374.48
99660	NCIDS II	p3	1	6	1.105	339.48
117028	NCIDS II	p3	1	4	4.06	398.30
121182	NCIDS II	p3	6	9	−2.303	266.26

nOHNH, number of hydrogen bond donors; nON, number of hydrogen bond acceptors; MW, molecular weight.

### Experimental Testing

**Figure 5:**
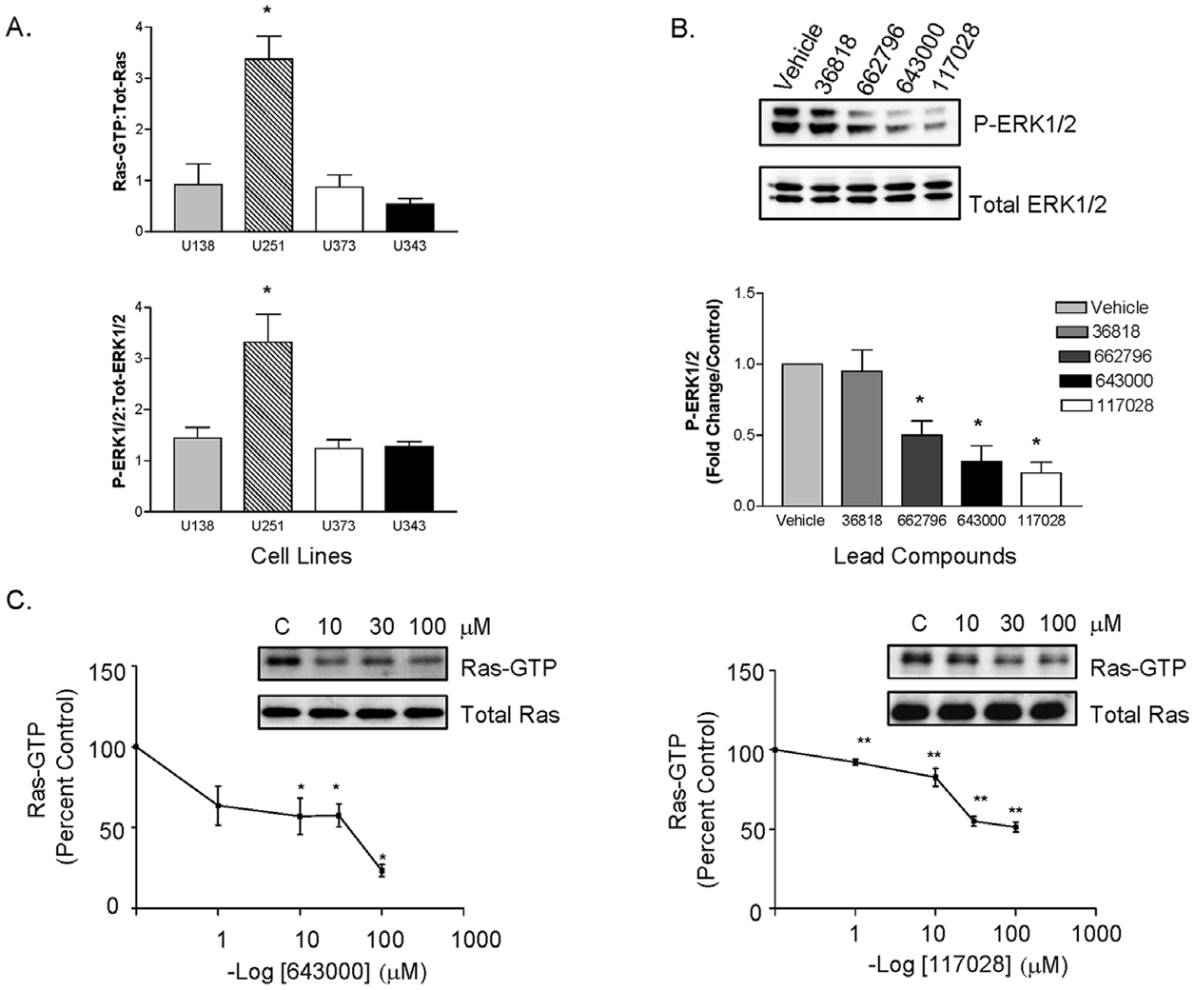
Experimental testing of candidate inhibitors. (*A,top*). The U138, U251, U373 and U343 glioblastoma cells were serum-starved for 48 hours and active Ras was precipitated with GST-fused Ras-binding domain of Raf-1 (Raf-1 RBD)-agarose beads and probed with anti-Ras antibody. *(A,bottom)* Lysates were immunoblotted for P-ERK1/2 or total ERK1/2. Data were quantitated and averaged from 4 independent experiments and presented as means ± SEM of the ratio of active Ras to total Ras *(top)* or the ratio of phosphorylated ERK1/2 to total ERK1/2 *(bottom)*. ^*^ *P*<0.05 for U251 vs U138, U373 and U343, one-way ANOVA. *(B)* Serum-starved U251 glioblastoma cells were treated with either vehicle (DMSO) control or 10 mM of the indicated compound for 24 hours. Cell lysates were immunoblotted for P-ERK1/2 or total ERK1/2. Immunoblots are representative of four independent experiments which were quantitated and shown graphically as means ± SEM; ^*^ *P*<0.05 vs. vehicle control, one-way ANOVA. *(C)* Cells (U251) were serum starved and subsequently treated with compound 643000 (left) or compound 117028 (right) at the indicated concentrations for 24 hours. Ras activity was assessed using the pull down assay. Immunoblots are representative of four independent experiments, which were quantitated and plotted as percent of vehicle control. Values are means ± SEM; ^*^ *P*<0.05 vs. vehicle control for compound 643000; ^**^ *P*<0.01 vs. vehicle control for compound 117028.

It is well known that Ras can activate the Raf/Mek/Erk signaling pathway. In order to select an appropriate cell line to evaluate the inhibitory properties of the candidate compounds *in vitro*, we determined basal Ras and ERK1/2 activity in several glioblastoma cell lines via Ras pull down assays and Western blot analysis, respectively. Results for four well characterized glioblastoma cell lines are shown in **Fig. 5A**. Among the lines examined, the U251 cells [50] were found to have the highest level of basal GTP bound Ras and this was associated with high basal downstream ERK1/2 phosphorylation (**Fig. 5A**). We employed this cell line to test the ability of the candidate inhibitor compounds to block the Ras-ERK pathway. We first determined the effect of each of four lead candidate compounds on ERK1/2 activation. Compounds were initially tested at a concentration of 10 µM and cells were treated for 24 hrs to allow adequate time to achieve decreases in steady state Ras and ERK activity levels. As shown in **Fig. 5B**, immunoblot analysis demonstrated that compounds 662796, 643000 and 117028 inhibited ERK1/2 phosphorylation in U251 glioblastoma cells. ERK1/2 phosphorylation levels from four separate experiments were quantitated and compounds 662796, 643000 and 117028 shown to elicit significant (50 to 75%) decreases in ERK1/2 activation. We next examined the ability of these compounds to decrease Ras activity in U251 cells (**Fig. 5C**). Ras pull down assays were performed to assess GTP bound (active) Ras. Two of the three compounds that suppressed ERK1/2 phosphorylation (643000 and 117028) also inhibited Ras activation in U251 cells. These data suggest that the ability of compounds 643000 and 117028 to decrease ERK1/2 phosphorylation derives from inhibition of Ras activity rather than nonspecific inhibitory effects on the ERK activation cascade downstream of Ras. We subsequently examined the dose dependence of Ras inhibition by compounds 643000 and 117028. Both compounds decreased basal Ras activity in a dose-dependent manner (**Fig. 5C**). For compound 64300, the half maximal inhibitory concentration appeared to be approximately 10–30 µM and nearly complete inhibition was achieved. For compound 117028, the maximal decrease achieved was approximately 40%. This may reflect the fact that there is a presence of highly active Ras guanine nucleotide exchange factors in this glioblastoma cell line. Nonetheless the ability of these non-optimized, computationally selected Ras inhibitory compounds to decrease the amount of constitutively active Ras, and its downstream target ERK, in this glioblastoma cell line suggests that this strategy holds considerable promise for limiting aberrant cancer cell growth mediated through this pathway.

### A proposed mechanism of action of the predicted ligands

The most prominent putative ligand binding site, p3, as well as its extension p3b, are located on lobe 2 near the C-terminus that leads to the membrane-interacting hypervariable region. Given our earlier MD and cell-biological studies that found that membrane-binding by the full length H-Ras [21], [22], [51] is allosterically modulated by the bound nucleotide, it is tempting to speculate that ligands targeting the predicted allosteric sites may interfere with Ras' attachment to the plasma membrane. It is also possible, however, that ligands that target these sites could allosterically modulate the conformation of the canonical switch region. This is consistent with the observed long-range coupled motions of loop 7 with the active site [23], [24], [43], [52]. Furthermore, a study combining crystallography, paramagnetic relaxation enhancement and calorimetry found that divalent ion-cyclen binding at a site equivalent to p3 allosterically stabilizes an effector-loop conformation with a weak potential for effector binding [53], [54]. Similarly, the current ligands may selectively stabilize GDP-like or transient structures and thereby decrease the pool of GTP-bound Ras through a population-shift mechanism [55]. Although further investigations are required to determine if the predicted ligands bind at the predicted sites, it is encouraging that they reduce ERK phosphorylation (activation) in U251 cells simultaneous with lowering the level of GTP-loaded Ras. Assuming that the compounds bind to Ras as predicted, we propose that they act by allosterically modulating membrane binding or, more likely, the catalytic site architecture.

Encouragingly, the micromolar anti-Ras activities observed in our initial experiments suggest that the predicted ligands may indeed serve as viable starting points for the lead generation against deregulated Ras signaling. Of special interest in terms of specificity are allosteric ligands targeting pocket p3 and p3b near the C-terminus and distal from the active site. The close proximity of these binding sites, and the fact that some compounds bind across multiple sites, provide an opportunity for linked-fragment drug design [56]. The bridging compounds can be used as scaffolds to discover new drugs with enhanced affinity and specificity. Obviously, the ligands identified in this work might not have high-affinity (tens of µM was required in our cell-based assays), or the required level of selectivity to be effective inhibitors. However, they can serve as crucial starting points for future development of new therapeutics against deregulated Ras signaling for which no selective inhibitors are available.

A major goal of the current work was to identify and validate novel drug binding sites on the Ras catalytic domain. Using a battery of computational techniques, that incorporate global conformational fluctuations into the hit identification process, we have shown that there are at least four potential allosteric pockets on the Ras catalytic domain. One of these novel sites near the C-terminus, termed p3, is of particular interest for inhibitor development. It is the most frequently targeted pocket both during our blind docking and VS exercises. That this pocket is involved in correlated motions with the active site [43] suggests that ligand binding at this pocket may allosterically modulate the active site architecture, as shown in other proteins [57], [58], [59]. This is consistent with the report that divalent ion-cyclen binding at p3 stabilizes a conformation that has weak effector-binding potential [54]. Thus, we propose that pocket p3 is a viable target against which leads can be generated to serve as starting points for the development and optimization of new drugs.

## Methods

### Structural ensemble generation

Available Ras crystallographic structures and MD simulation snapshots (see below) were analyzed using the Bio3D package [36]. Details of this procedure have been described previously [23], [24], briefly, structural superposition was performed on the invariant “core” as defined by Grant et al. [36]. Next, the Cartesian coordinates of equivalent Cα atoms were used to define the elements of a covariance matrix. The covariance matrix was then diagonalized to derive principal components with their associated variances. The crystallographic and MD conformers were projected into the sub-space defined by PC1-3 where the maximum variation of the conformational distribution was observed (see [23], [24] for further details). Structures then underwent average-linkage hierarchical clustering according to the pairwise distances obtained from their projection onto the first three principal components obtained from analysis of available crystal structures. Clustering based on pairwise Cα atom RMSD yielded similar major clusters. Inspection of the resulting dendogram was used to partition structures into seven to eight dominant groups (ranked according to their populations). The closest structure to the average structure from each cluster, in terms of RMSD, was chosen as a representative for further binding site mapping and virtual screening analysis.

### Identification of novel binding sites

We used the FTMap method of Brenke and co-workers to highlight regions on the Ras catalytic domain surface that have the potential to bind the highest number of small molecular probes [42]. Both crystal structures and each cluster representative from MD were subject to fragment mapping. Hot-spot residues (those that comprise prominent fragment binding sites) across all structures were analyzed with the Bio3D package. A residue was assumed to be in contact with a probe molecule if any two heavy atoms from the probe and residue were within 5.0 Å.

To further characterize novel binding sites we used AutoLigand from the AutoDockTools (ADT) package [44]. AutoLigand predicts possible ligand-binding sites by searching for a contiguous envelope with the specified volume of 70 favorable energy points that represent potential atomic centers for ligand atoms. We applied AutoLigand to scan each conformer in the structural ensemble. High affinity binding pockets in each of the Ras conformers were identified and the common residues lining those sites were determined using Bio3D. As a positive control, AutoLigand and FTMAP both successfully identified the nucleotide-binding site in all ensemble conformers.

An additional “blind docking” ligand based search with known Ras binders in public databases was used to determine if there are sites on Ras that are frequently visited by a library of small molecule compounds. It was shown that such blind docking procedures correctly identify binding sites in 80% of cases but fail to discriminate between true ligands and decoys [60]. We used AutoDock 4.2 [61] to “blind dock” a library of 267 putative ligands from the PubChem Bioassy database. These compounds were shown to bind to Ras at unknown sites. The PubChem Bioassy database has potential Ras inhibitors comprising 267 compounds as determined by a high throughput fluorescent-GTP binding assay (AID database accession code: 759). Compounds inducing at least 20% reduction in fluorescence relative to the ligand-free control were termed potential inhibitors in the high throughput assay. For our VS study, the ligands were prepared with ADT by adding polar hydrogen atoms and calculating the Gasteiger charges and torsions.

AutoDock 4.2 was used to dock the ligands onto the entire surface of the K-Ras crystal structure (PDB code 3GFT) with a grid dimension large enough to cover the entire protein (143×149×155 Å, with 0.375 Å grid spacing). The bound GTP-analogue phosphoaminophosphonic acid-guanylate ester (GNP) was kept to exclude ligands that may target the catalytic site. A population size of 400, a 2 Å cutoff for clustering, 1.5×10^8^ energy evaluations and 256 hybrid Lamarckian genetic algorithm runs were used. The predicted poses were analyzed in terms of their potential to form hydrogen bonds and van der Waals contacts with the protein. Hydrogen bonds were defined by a donor-acceptor distance of less than 3.5 Å and a donor-hydrogen-acceptor angle between 150° and 180°. Van der Waals contacts were defined by a carbon-carbon distance cutoff of 5.0 Å. The relative importance of target-sites was determined by ranking the docked compounds according to their empirical inhibition constant after eliminating compounds that did not consistently bind at the same site and those violating Lipinski's rule [62]. Pocket volumes were calculated with Povme (POcketVolumeMEasurer) program [63] by first aligning all ensemble conformers.

### Molecular dynamics simulation

Simulations of K-Ras were carried out with bound Mg^2+^GTP and Mg^2+^GDP, both modeled from a high-resolution crystal structure of K-Ras (PDB code 2PMX). Protonation states for all titratable residues were determined using PDB2PQR [64]. The LEaP module of AMBER 10 package [65] was used for the addition of missing hydrogen atoms. The systems were neutralized by addition of counter ions at pH 7 and solvated with TIP3P waters with the buffering distance of 10 Å. All simulations were performed with the AMBER 10 package [65] and the ff99SB force field [66] and previously developed parameters for guanine nucleotides [67].

Energy minimization was carried out with decreasing constraints on the heavy atom positions, followed by constant volume heating to 300 K for ∼10 ps, and a 200ps constant temperature and constant pressure (1 atm) equilibration. The production phase involved multi-copy unrestrained runs for enhanced sampling and improved statistics. Three independent 20 ns production runs with different initial velocities were carried out on each system resulting in a cumulative simulation time of 120 ns. The integration time step was 2 fs with bonds involving hydrogen atoms restrained by SHAKE [68]. Short-range non-bonded interactions were truncated at 10 Å while long-range electrostatic interactions were evaluated with the Particle-Mesh Ewald method [69]. All simulation analysis was performed with the Bio3D package.

### Virtual screening

Compounds in the NCIDS II and ZINC drugs-now subset satisfying a Tanimoto score threshold of 60% were docked into each of the seven and eight representative conformers of the crystallographic- and MD-ensembles, respectively. Each protein conformer was first prepared with the Protein Preparation Wizard of the Schrodinger program suite (**Fig. S4**). Bond orders were assigned, and hydrogen bond networks, rotating hydroxyl and thiol hydrogens were optimized. A grid enclosing each binding site of interest was generated using Receptor Grid Generation.

Each compound was prepared using Ligprep of Schrodinger using the OPLS_2005 force field at pH 7.0+−2.0 with ionization states generated by Epik 1.6. To enhance ligand diversity, for each ligand up to 32 stereoisomers and 8 low energy ring conformations were generated, resulting in a total of 5,267 and 39,490 3D structures for NCIDSII and ZINC, respectively. Then triple steps virtual screenings, encompassing high throughput virtual screening, standard precision, and extra precision were performed using Glide [47], [48].

### Re-scoring

Each docked pose was re-scored with Accelrys Discovery Studio using PLP(1 and 2) [70], Jain [71], PMF [72], PMF04 [73] and Ludi (1, 2, and 3) [74], [75], [76] scoring functions. Although absolute scores given by the different scoring methods cannot be compared, the relative rankings of each compound with the different scoring methods provide useful information. Thus, compounds ranked as top 20 with each scoring function were combined to build a new library for each binding site. Visual analysis and filtering for drug-likeness by Lipinski's rule of fives [62], [77] (no more than five hydrogen bond donors, 10 hydrogen bond acceptors, low molecular weight not greater than 500 g/mol, and a logP not greater than 5), was applied to further reduce the number of compounds suggested for experimental testing.

### ERK1/2 Phosphorylation Assay

Glioblastoma cells were serum-starved for 24 hours in DMEM containing 0.1% BSA and subsequently treated or not for 24 hours with compounds obtained from the NCIDS II and ZINC small molecule database. Cells were washed and lysed in buffer containing 50 mM Tris HCL (pH 7.4), 100 mM NaCl, 5 mM MgCl_2_, 0.1% Triton X100, 10% glycerol, 1 mM phenylmethylsulfonylfluoride, 1 mM DTT, 0.1 mM sodium orthovanadate, 10 µg/mL leupeptin, and 10 mg/mL aprotinin. Lysates were clarified by centrifugation and protein content was measured. Cell lysates were solubilized by boiling for 10 minutes in Leammli buffer. Proteins were resolved by SDS-PAGE analysis and membranes were probed with an anti-phospho-ERK1/2 antibody (Cell Signaling). Total ERK1/2 was determined by immunoblotting with anti-ERK1/2 polyclonal antibody (Cell Signaling). Proteins were visualized using enhanced chemiluminescence and quantitated using gel documentation software (Alpha Innotech Corp., San Leandro, CA).

### Ras Pull-Down Assay

Ras activation assays were performed following the affinity precipitation protocol provided by the manufacturer (RAs pull-down assay kit: Upstate Biotechnology lake Placid, NY). Briefly, lysates were incubated with Raf-1 RBD for 45 min at 4°C and then centrifuged to pellet the agarose beads. Agarose beads were washed, and pellets were resuspended in 2× Laemmli sample buffer and boiled for 5 min. The supernatant was collected and cellular proteins were resolved by SDS-PAGE and analyzed by immunoblotting with an anti-Ras antibody (Cell Signaling).

### Statistical Analysis

Statistical analysis was determined using ANOVA followed by the Tukey *multicomparison* test. A value of *P*<0.05 was considered significant.

## Supporting Information

Figure S1 **Heat map clustering of Ras structures in the PC1 to PC3 planes.** Structure labels are colored by nucleotide state (red for GTP and green for GDP). Major conformational groupings are indicated by the orange labels and corresponding marginal dendrograms (see text for details).
(TIF)

Figure S2 **Pocket p1 dynamics. Residues lining P1 (see** **Table 1** **) are shown in red surface.** Tyr71 (magenta) re-orients to enable access to P1. Probability density of Chi1 side chain dihedral angles of Tyr 71 shows distinct orientations.
(TIF)

Figure S3 **Projection of representative conformers (red) of K-Ras MD ensemble (blue) on the first two dominant principal components obtained from the analysis of crystallographic ensemble.** MD conformers lie in between two major crystal clusters (gray) associated with GTP-bound (PC1: −7 to 0) and GDP-bound (PC1: 15 to 20). The representative conformers were identified based on RMSD and PCA based clustering, see text for details.
(TIF)

Figure S4 **A multi-level computational approach for the identification of small molecules that bind to novel allosteric sites on Ras. MD, molecular dynamics; NCIDS II, National Cancer Institute diversity set II; HTVS, high throughput virtual screening; SP, standard precision; XP, extra precision.**
(TIF)

Table S1 **Representative structure obtained from analyzing the Ras crystallographic ensemble.**
(DOC)

Movie S1 **Ensemble fragment mapping results highlight three non-nucleotide binding sites.** Representative Ras crystal structure conformers (gray protein cartoon) are shown along with the nucleotide-binding site (red molecular surface representation) and new potential binding sites p1, p2 and p3 (in pink, green and blue molecular surface representations respectively). Also shown is an average Ras conformer where relative chain thickness and color scale (red: high, gray: low) represent residue wise probe occupancy values (see text for details).
(MP4)
