# Supplementary figures and images for: Novel Allosteric Sites on Ras for Lead Generation
Source: PLoS One. 2011 Oct 25;6(10):e25711. doi: 10.1371/journal.pone.0025711 (PMC3201956; doi:10.1371/journal.pone.0025711)

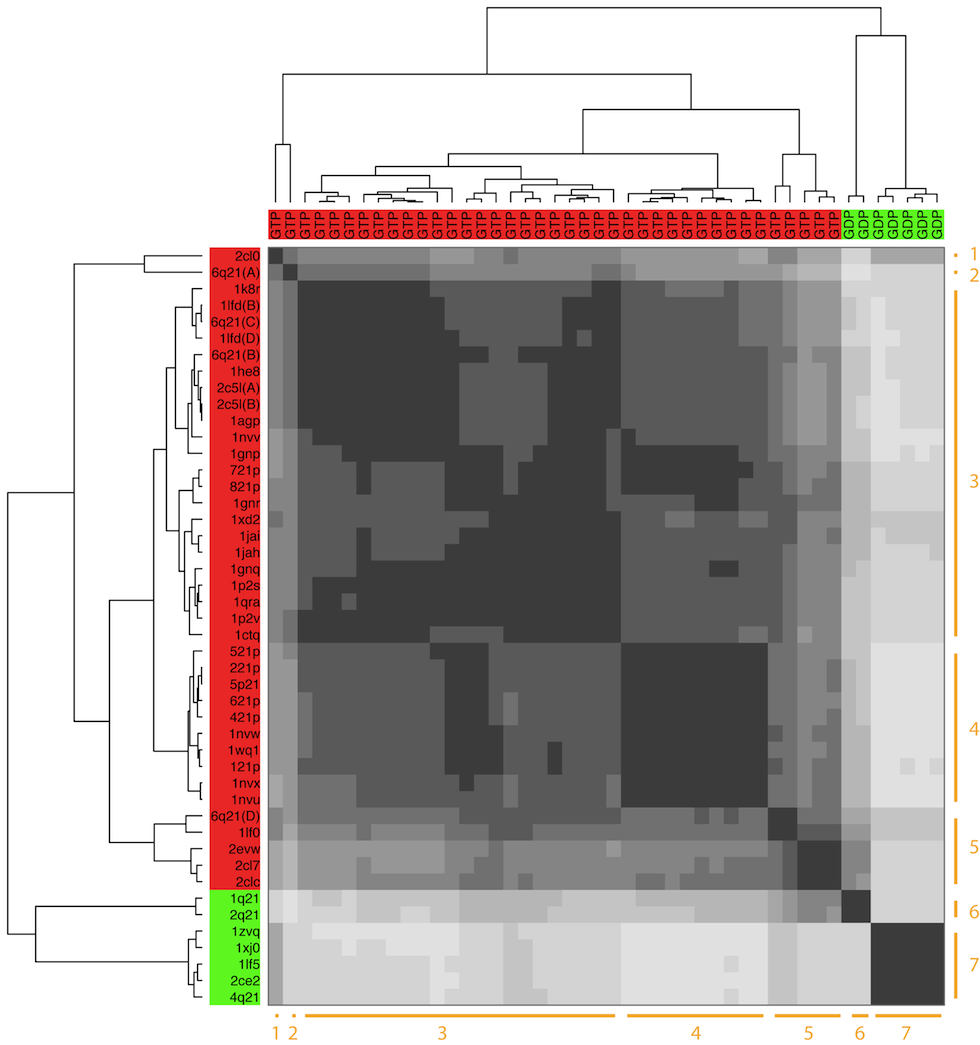

Supplement: Figure S1 — Heat map clustering of Ras structures in the PC1 to PC3 planes. Structure labels are colored by nucleotide state (red for GTP and green for GDP). Major conformational groupings are indicated by the orange labels and corresponding marginal dendrograms (see text for details). (TIF) [file pone.0025711.s001.tif]

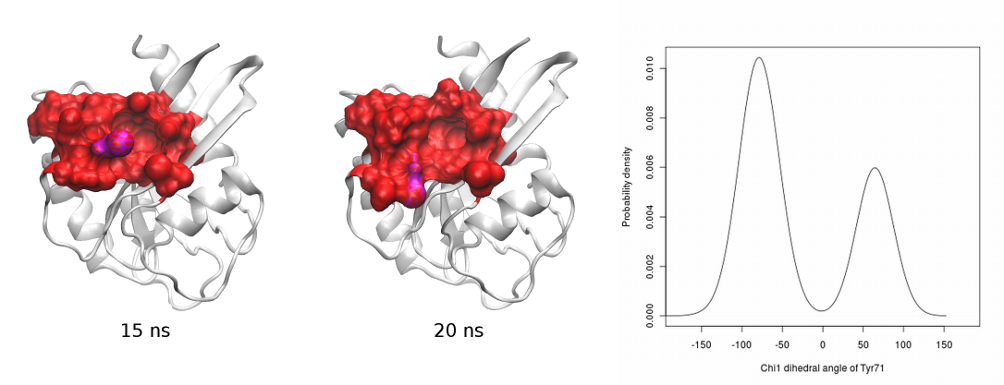

Supplement: Figure S2 — Pocket p1 dynamics. Residues lining P1 (see Table 1 ) are shown in red surface. Tyr71 (magenta) re-orients to enable access to P1. Probability density of Chi1 side chain dihedral angles of Tyr 71 shows distinct orientations. (TIF) [file pone.0025711.s002.tif]

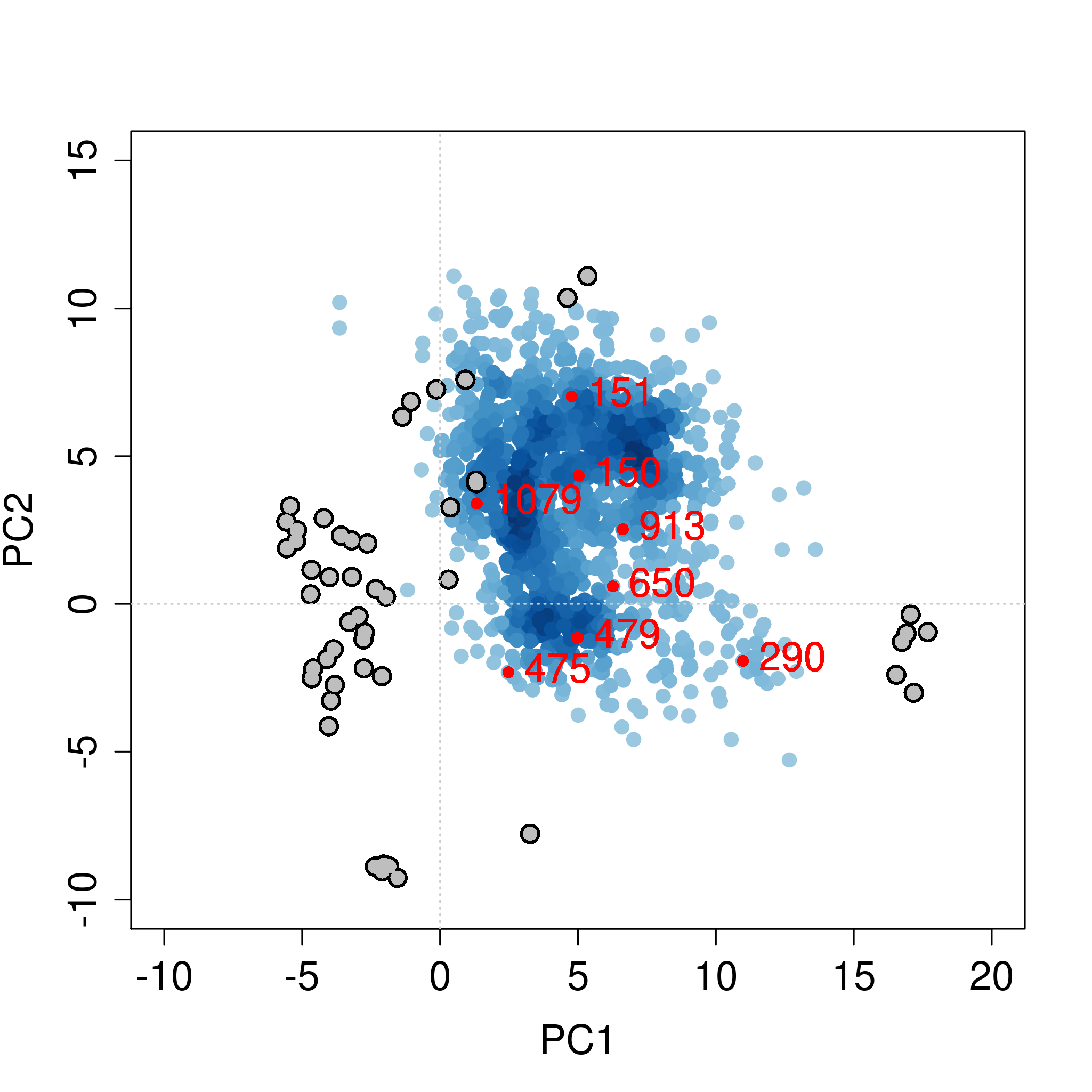

Supplement: Figure S3 — Projection of representative conformers (red) of K-Ras MD ensemble (blue) on the first two dominant principal components obtained from the analysis of crystallographic ensemble. MD conformers lie in between two major crystal clusters (gray) associated with GTP-bound (PC1: −7 to 0) and GDP-bound (PC1: 15 to 20). The representative conformers were identified based on RMSD and PCA based clustering, see text for details. (TIF) [file pone.0025711.s003.tif]

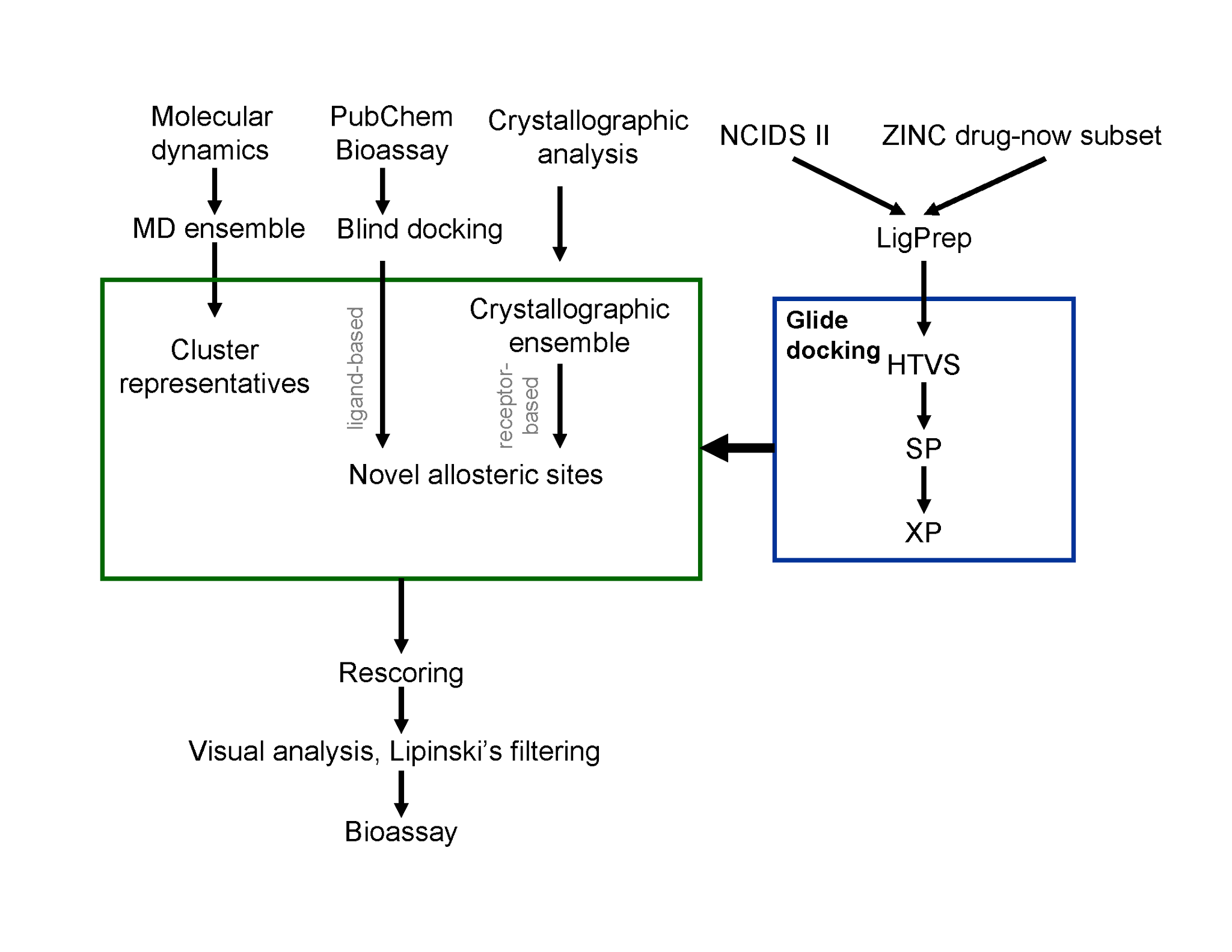

Supplement: Figure S4 — A multi-level computational approach for the identification of small molecules that bind to novel allosteric sites on Ras. MD, molecular dynamics; NCIDS II, National Cancer Institute diversity set II; HTVS, high throughput virtual screening; SP, standard precision; XP, extra precision. (TIF) [file pone.0025711.s004.tif]
