# Supplementary material for: Novel Allosteric Sites on Ras for Lead Generation
Source: PLoS One. 2011 Oct 25;6(10):e25711. doi: 10.1371/journal.pone.0025711 (PMC3201956; doi:10.1371/journal.pone.0025711)
Supplement: Table S1 — Representative structure obtained from analyzing the Ras crystallographic ensemble. (DOC) [file pone.0025711.s005.doc]

**Novel Allosteric Sites on Ras for Lead Generation**

Barry J. Grant ^1,*^, Suryani Lukman^2 ,3,*^, Harrison J. Hocker ^4^, Jaqueline Sayyah ^5^,

Joan Heller Brown ^5^, J. Andrew McCammon ^1,5^ and Alemayehu A. Gorfe ^4^

^1^ Department of Chemistry and Biochemistry, Center for Theoretical Biological Physics and Howard Hughes Medical Institute, University of California San Diego, La Jolla, California, USA

^2^ Bioinformatics Institute, Agency for Science, Technology and Research, Singapore

^3^ Department of Chemistry, University of Cambridge, UK

^4^ Department of Integrative Biology and Pharmacology, University of Texas Health Science Center at Houston, Texas, USA

^5^ Department of Pharmacology, University of California San Diego, La Jolla, California, USA

*These authors contributed equally to this work.

E-mails: bgrant@mccammon.ucsd.edu, sl471@cam.ac.uk, Alemayehu.G.Abebe@uth.tmc.edu

**Table S1**. Representative structure obtained from analyzing the Ras crystallographic ensemble.

| **PDB ID** | **Resolution (Å)** | **Source** | **Ligand** | **Isoform** |
| --- | --- | --- | --- | --- |
| 1LF0 | 1.7 | *Homo Sapiens* | GNP | H |
| 2PMX | 2.3 | *Homo Sapiens* | GNP | K |
| 1QRA | 1.6 | *Homo Sapiens* | GTP | H |
| 1WQ1 | 2.5 | *Homo Sapiens* | GDP, AlF3 | H |
| 2Q21 | 2.2 | *Homo Sapiens* | GDP | H |
| 2RGD | 2.0 | *Homo Sapiens* | GNP | H |
| 4Q21 | 2.0 | *Homo Sapiens* | GDP | H |
